# Continuing Professional Development ‐ Medical Imaging

**DOI:** 10.1002/jmrs.827

**Published:** 2024-09-20

Maximise your CPD by reading the following selected article and answer the five questions. Please remember to self‐claim your CPD and retain your supporting evidence. Answers will be available via the QR code and published in JMRS – Volume 72, Issue 4, December 2025.

## Medical Imaging – Original Article

### Preliminary image evaluation performance of radiographers in one New Zealand District: a 6‐month prospective study

Lewis K , Mdletshe S , Doubleday A , Pieterse T. (2024). J Med Radiat Sci 10.1002/jmrs.810 PMC1163835739186544

1. Which of the following x‐ray examinations from the participating emergency departments (ED) were included in this study?
  - All x‐ray imaging that resulted from trauma
  - All appendicular x‐ray imaging from all pathologies
  - All appendicular x‐ray imaging that resulted from trauma only
  - All appendicular x‐ray imaging that resulted from trauma within 14 days of presentation
2. In this study, what was the most missed abnormality?
  - Avulsion fractures in the finger
  - Neck of femur fracture
  - Dislocated shoulder
  - Spiral fracture of the tibia
3. What was a key finding from the April study results?
  - Participants were not completing preliminary image evaluation (PIE) comments on difficult studies
  - PIE comments were too wordy, lowering their accuracy
  - The prevalence of abnormalities was lower than anticipated
  - There were very few PIE comments made
4. Which of the following types of bias can cause an unusually high abnormality prevalence?
  - Case selection bias
  - Spectrum bias
  - Verification bias
  - Observer bias
5. According to this study, which of the following is recommended for radiographers participating in a PIE system to maintain their performance in detecting and describing abnormalities on acute extremity x‐ray examinations?
  - A pay increase
  - Additional time to review the x‐ray examination
  - Workplace‐provided reference documents and textbooks
  - Training and ongoing feedback

## Answers

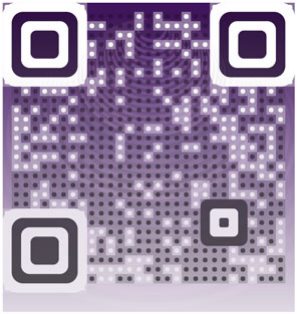

Scan this QR code to find the answers.
